# Supplementary material for: Performance of amplicon and capture based next-generation sequencing approaches for the epidemiological surveillance of Omicron SARS-CoV-2 and other variants of concern
Source: PLoS One. 2024 Apr 29;19(4):e0289188. doi: 10.1371/journal.pone.0289188 (PMC11057745; doi:10.1371/journal.pone.0289188)
Supplement: S1 File — (DOCX) [file pone.0289188.s001.docx]

**S1 File. Custom protocol for RT-PCR amplification of SARS-CoV-2 RNA.** The protocol was used for manual library preparation.

**Step 1: Annealing of random Hexamers to RNA**

**Reagents required**

Random Hexamers 50 μM (Invitrogen, California, USA)

dNTP mix 10 mM (Invitrogen, California, USA)

**Reaction Mix**

| Reagent | Volume (μl) |
| --- | --- |
| Sample RNA | 11 |
| Random Hexamers | 1 |
| dNTPs | 1 |

**Thermocycler program**

65 ºC 5’

**Step 2: Retrotranscription into cDNA**

**Reagents required**

RNaseOUT™ Ribonuclease Inhibitor (Recombinant) 40 U/ul (Invitrogen, California, USA)

SuperScript™ IV Reverse Transcriptase 200 U/μl Kit (Invitrogen, Vilnius, Lithuania)

**Reaction Mix**

| Reagent | Volume (μl) |
| --- | --- |
| SSIV Buffer5X | 4 |
| DTT 100mM | 1 |
| RNAse OUT 40 U/ul | 1 |
| SSIV Reverse Transcriptase 200U/μl | 1 |
| Mix 1 product | 13 |

**Thermocycler program**

42 ºC 50’

70 ºC 10’

**Step 3: Amplification of cDNA with ARTIC v3 primer pools**

**Reagents required**

Q5 HotStart High-Fidelity DNA Polymerase Kit (New England Biolabs, Massachusetts, USA)

Primer pools ARTIC v3 (pool 1 & pool 2) (Integrated DNA technologies, Iowa, USA)

dNTP mix 10 mM (Invitrogen, California, USA)

**Reaction Mix**

| Reagent | Mix Pool 1 (μl) | Mix Pool 2 (μl) |
| --- | --- | --- |
| Q5 reaction Buffer 5X | 5 | 5 |
| dNTPs 10mM | 0,5 | 0,5 |
| Q5 HotStart DNA Polymerase2 U/μl | 0,25 | 0,25 |
| ARTIC Panel Pool 1 10 mM | 4 |  |
| ARTIC Panel Pool 2 10 mM |  | 4 |
| Nuclease-Free Water | 12,75 | 12,75 |
| cDNA from Mix 2 | 2,5 | 2,5 |

**Thermocycler program**

| Temperture | Cycles |
| --- | --- |
| 98 ºC 3’ | 1 |
| 98 ºC 15” | 30 |
| 72 ºC 5’ |  |
| 4 ºC Hold | 1 |
