# Supplementary material for: Performance of amplicon and capture based next-generation sequencing approaches for the epidemiological surveillance of Omicron SARS-CoV-2 and other variants of concern
Source: PLoS One. 2024 Apr 29;19(4):e0289188. doi: 10.1371/journal.pone.0289188 (PMC11057745; doi:10.1371/journal.pone.0289188)
Supplement: S1 Dataset — (DOCX) [file pone.0289188.s004.docx]

All genetic sequences analysed for this study have been published in the GISAID database, with the accession numbers indicated in the table. Two sequences were generated per sample, one for the amplicon method and another one for the capture method.

| **ID** | **GISAID_Amplicon** | **GISAID_Capture** |
| --- | --- | --- |
| 1 | EPI_ISL_17621823 | EPI_ISL_17543953 |
| 2 | EPI_ISL_17621824 | EPI_ISL_17543954 |
| 3 | EPI_ISL_17621825 | EPI_ISL_17543955 |
| 4 | EPI_ISL_17621826 | EPI_ISL_17543956 |
| 5 | EPI_ISL_17621827 | EPI_ISL_17543957 |
| 6 | EPI_ISL_17621829 | EPI_ISL_17543958 |
| 7 | EPI_ISL_17621830 | EPI_ISL_17543959 |
| 8 | EPI_ISL_17621831 | EPI_ISL_17543960 |
| 9 | EPI_ISL_17621832 | EPI_ISL_17543961 |
| 10 | EPI_ISL_17621833 | EPI_ISL_17543962 |
| 11 | EPI_ISL_17621834 | EPI_ISL_17543963 |
| 12 | EPI_ISL_17621835 | EPI_ISL_17543964 |
| 13 | EPI_ISL_17621836 | EPI_ISL_17543965 |
| 14 | EPI_ISL_17621837 | EPI_ISL_17543966 |
| 15 | EPI_ISL_17621838 | EPI_ISL_17543967 |
| 16 | EPI_ISL_17621839 | EPI_ISL_17543968 |
| 17 | EPI_ISL_17621840 | EPI_ISL_17543969 |
| 18 | EPI_ISL_17621842 | EPI_ISL_17543970 |
| 19 | EPI_ISL_17621843 | EPI_ISL_17543971 |
| 20 | EPI_ISL_17621844 | EPI_ISL_17543972 |
| 21 | EPI_ISL_17621868 | EPI_ISL_17543996 |
| 22 | EPI_ISL_17621869 | EPI_ISL_17543997 |
| 23 | EPI_ISL_17621872 | EPI_ISL_17544000 |
| 24 | EPI_ISL_17621873 | EPI_ISL_17544001 |
| 25 | EPI_ISL_17621874 | EPI_ISL_17544002 |
| 26 | EPI_ISL_17621875 | EPI_ISL_17544003 |
| 27 | EPI_ISL_17621876 | EPI_ISL_17544004 |
| 28 | EPI_ISL_17621877 | EPI_ISL_17544005 |
| 29 | EPI_ISL_17621878 | EPI_ISL_17544006 |
| 30 | EPI_ISL_17621879 | EPI_ISL_17544007 |
| 31 | EPI_ISL_17621880 | EPI_ISL_17544008 |
| 32 | EPI_ISL_17621882 | EPI_ISL_17544010 |
| 33 | EPI_ISL_17621883 | EPI_ISL_17544011 |
| 34 | EPI_ISL_17621884 | EPI_ISL_17544012 |
| 35 | EPI_ISL_17621885 | EPI_ISL_17544013 |
| 36 | EPI_ISL_17621886 | EPI_ISL_17544014 |
| 37 | EPI_ISL_17621887 | EPI_ISL_17544015 |
| 38 | EPI_ISL_17621888 | EPI_ISL_17544016 |
| 39 | EPI_ISL_17621889 | EPI_ISL_17544017 |
| 40 | EPI_ISL_17621890 | EPI_ISL_17544018 |
| 41 | EPI_ISL_17621891 | EPI_ISL_17544019 |
| 42 | EPI_ISL_17621892 | EPI_ISL_17544020 |
| 43 | EPI_ISL_17621893 | EPI_ISL_17544021 |
| 44 | EPI_ISL_17621894 | EPI_ISL_17544022 |
| 45 | EPI_ISL_17621895 | EPI_ISL_17544023 |
| 46 | EPI_ISL_17621909 | EPI_ISL_17544037 |
| 47 | EPI_ISL_17621910 | EPI_ISL_17544038 |
| 48 | EPI_ISL_17621911 | EPI_ISL_17544039 |
| 49 | EPI_ISL_17621912 | EPI_ISL_17544040 |
| 50 | EPI_ISL_17621913 | EPI_ISL_17544041 |
| 51 | EPI_ISL_17621914 | EPI_ISL_17544042 |
| 52 | EPI_ISL_17621841 |  |
| 53 | EPI_ISL_17621881 | EPI_ISL_17544009 |
| 54 | EPI_ISL_17621828 |  |
| 55 | EPI_ISL_17621845 | EPI_ISL_17543973 |
| 56 | EPI_ISL_17621846 | EPI_ISL_17543974 |
| 57 | EPI_ISL_17621847 | EPI_ISL_17543975 |
| 58 | EPI_ISL_17621848 | EPI_ISL_17543976 |
| 59 | EPI_ISL_17621849 | EPI_ISL_17543977 |
| 60 | EPI_ISL_17621850 | EPI_ISL_17543978 |
| 61 | EPI_ISL_17621851 | EPI_ISL_17543979 |
| 62 | EPI_ISL_17621852 | EPI_ISL_17543980 |
| 63 | EPI_ISL_17621853 | EPI_ISL_17543981 |
| 64 | EPI_ISL_17621854 | EPI_ISL_17543982 |
| 65 | EPI_ISL_17621855 | EPI_ISL_17543983 |
| 66 | EPI_ISL_17621856 | EPI_ISL_17543984 |
| 67 | EPI_ISL_17621857 | EPI_ISL_17543985 |
| 68 | EPI_ISL_17621858 | EPI_ISL_17543986 |
| 69 | EPI_ISL_17621859 | EPI_ISL_17543987 |
| 70 | EPI_ISL_17621860 | EPI_ISL_17543988 |
| 71 | EPI_ISL_17621861 | EPI_ISL_17543989 |
| 72 | EPI_ISL_17621862 | EPI_ISL_17543990 |
| 73 | EPI_ISL_17621863 | EPI_ISL_17543991 |
| 74 | EPI_ISL_17621864 | EPI_ISL_17543992 |
| 75 | EPI_ISL_17621865 | EPI_ISL_17543993 |
| 76 | EPI_ISL_17621866 | EPI_ISL_17543994 |
| 77 | EPI_ISL_17621867 | EPI_ISL_17543995 |
| 78 | EPI_ISL_17621870 | EPI_ISL_17543998 |
| 79 | EPI_ISL_17621871 | EPI_ISL_17543999 |
| 80 | EPI_ISL_17621896 | EPI_ISL_17544024 |
| 81 | EPI_ISL_17621897 | EPI_ISL_17544025 |
| 82 | EPI_ISL_17621898 | EPI_ISL_17544026 |
| 83 | EPI_ISL_17621899 | EPI_ISL_17544027 |
| 84 | EPI_ISL_17621900 | EPI_ISL_17544028 |
| 85 | EPI_ISL_17621901 | EPI_ISL_17544029 |
| 86 | EPI_ISL_17621902 | EPI_ISL_17544030 |
| 87 | EPI_ISL_17621903 | EPI_ISL_17544031 |
| 88 | EPI_ISL_17621904 | EPI_ISL_17544032 |
| 89 | EPI_ISL_17621905 | EPI_ISL_17544033 |
| 90 | EPI_ISL_17621906 | EPI_ISL_17544034 |
| 91 | EPI_ISL_17621907 | EPI_ISL_17544035 |
| 92 | EPI_ISL_17621908 | EPI_ISL_17544036 |
| 93 | EPI_ISL_17621915 | EPI_ISL_17544043 |
| 94 | EPI_ISL_17621916 | EPI_ISL_17544044 |
